# The Influence of Users’ Spatial Familiarity on Their Emotional Perception of Space and Wayfinding Movement Patterns

**DOI:** 10.3390/s21082583

**Published:** 2021-04-07

**Authors:** Ju Yeon Kim, Jin Kyung Choi, Won Hee Han, Jong Ha Kim

**Affiliations:** 1 Department of Interior Architectural Design, Soongsil University, 360 Sando-ro, Dongjak-Gu, Seoul 06978, Korea; kjy@ssu.ac.kr (J.Y.K.); durslw@ssu.ac.kr (J.K.C.); wonheepaulhan@ssu.ac.kr (W.H.H.); 2 Department of Architecture and Fire Safety, Dongyang University, 145, Dongyangdae-ro, Punggi-eup, Yeongju-si 36040, Korea

**Keywords:** environmental psychology, emotional perception, wayfinding, gender, spatial familiarity

## Abstract

In order to evaluate the sensory perceptions of users who visited a train station, this study aimed to conduct an evaluation of their spatial emotions and identify the distance and type of transfer. For evaluation and verification, emotional recognition and wayfinding types were analyzed according to types in the groups (gender, age, and spatial familiarity) of experimental participants. There were two research questions: “Will the length of movement patterns in the experiment environment vary depending on the types of the participant group?” and “Is there any moderating effect in the interaction between spatial familiarity and the types of the participant groups?” A total of 28 participants were recruited with consideration of gender, age, and familiarity with spatial experience, which were used to analyze the participant groups. The experiment was conducted at a train station, and a vignette was presented to the participants to record the route and pattern of their wayfinding, followed by providing a questionnaire to record their spatial perception. SPSS was used to conduct a T-test, factor analysis, and multidimensional scaling (MDS). The differences in spatial perception were arranged in visual positioning based on emotional vocabulary, and average movement distances in the participant groups were compared in accordance with the type of wayfinding and interaction effect by ANOVA. The results showed that there was a difference in spatial perception depending on the negative emotional vocabulary and type of participant. An emotional positioning map for average comparison was prepared for each participant group (gender, age, and spatial familiarity) by using the factors extracted in the factor analysis (emotional factor, management factor, and aesthetic factor). Female and unfamiliar groups displayed negative results in the emotional factor (F = 7.202, *p* < 0.05). In addition, male and familiar groups displayed negative results in the management factor (F = 3.058, *p* < 0.10). In wayfinding, there was an interaction between gender and the resident group based on the status of their spatial familiarity. Through this, it was possible to extract negative emotional evaluations according to the type of participant and the interaction factors for the type and length of the wayfinding.

## 1. Introduction

### 1.1. Research Background and Purpose

Wayfinding is the act of starting from a specific point and arriving at a destination. It refers to a series of cognitive processes that involve the abilities of understanding the surrounding environment, establishing a plan, converting it into a behavioral activity, and implementing the necessary decision at each moment [1]. Therefore, recognizing the surrounding environment in wayfinding has an important meaning as the first step. Most behaviors made by people take place in the context of their environment. More than 80% of human spatial perception is collected by vision, which is imaged and stored in the cerebrum [2], and environmental information acquired in spatial perception becomes an important element of wayfinding. Since the visual information acquired in wayfinding becomes background information, those people who have information can much more easily arrive at their destination than first-time visitors who have little or no information.

Bell, et al. [3] defined environmental psychology as the psychological state that people feel in a specific environment among various living environments. This allows studies on the process and outcome of how humans interact with the environment, and affecting each other [4]. Human decision-making in wayfinding, influenced by environmental psychology, is easy in a well-arranged space, but it is not expected to be easy in an environment where various signs, shops, and crowds are intricately intertwined. Accordingly, studies are being conducted on achieving effective wayfinding in crowded spaces, such as commercial spaces and public spaces that cause confusion to users while wayfinding [4,5,6,7,8,9,10] and in spaces that cause confusion for users, such as public transportation, etc. [11,12]. Efficient planning for users is important in public transportation [13], and In and Choe [14] conducted a study on a subway space, which is a representative public transportation method. It was confirmed that in complicated subway transfer sections (Sindorim Station, Seoul), factors that make wayfinding difficult include a lack of information transmission of the visual information system that provides information to users and its complex arrangement, etc. [15]. Thus, a complex space should be designed in such a way that it allows humans to easily understand space and accurately recognize spatial information. Such recognition of environment is expressed as an description of feelings of space experience, and analysis of those expressed feelings may lead to an understanding of the psychological response that humans have to space.

As in previous studies evaluating the perception of spatial experience with emotional vocabulary, An, Kang, and Lee [16] suggested two paths in a large shopping mall (COEX Mall), including the main walking path, to give the participants a preferred choice of path. After making that choice, emotional vocabulary evaluation for the selected route was conducted. A total of 16 pairs were used for the evaluation of emotional vocabulary, and the study conducted on a 7-point Likert scale for bipolar adjectives. Kim and Joo [17] presented the image of a sign to the participants to evaluate the design satisfaction with the guide sign, and then they conducted a 5-point scale evaluation using the already selected 7 pairs of adjectives. Kim and Park [18] conducted an eye-tracking experiment and post-emotional vocabulary evaluation to examine what kind of gaze characteristics visitors show when they look at the street. The goal was to improve the streetscape environment. The stimulus of a horizontal landscape was also presented to measure the gaze characteristics, and the emotional vocabulary of 17 pairs of adjectives on a 10-point scale measured the sensibility of the stimulus. Kim et al. [4] conducted a study to activate street space, focusing on pedestrians using that street space. A gaze-behavioral observation experiment was conducted to examine each pedestrian’s gaze and behavioral characteristics in that street space. After the experiment, an emotional vocabulary evaluation was conducted for the test subject area. Emotional scales for a total of 16 emotional vocabularies were evaluated for five levels of emotions. Jeon [19] conducted a study on how the stress factors, and stress factors of the wayfinding process, at subway transfer stations affect the place satisfaction of the participants. The experimental environment was created using a virtual reality program, and after a pathfinding experiment in that virtual reality space, a 5-point Likert scale was use to evaluate 20 emotional vocabularies. In addition to extracting the physical method for wayfinding through observational research, i.e., a research method in behavioral science, the extraction of psychological expression of emotions is considered to be an important interpretation of such data.

The train station selected as the experiment target site in this study is the main station in Suwon, Korea. For actual visitors, this station was selected as a difficult place to find destinations, such as transfers and exits, due to the structural problems of the train station. In particular, after opening in 2003, this station added different facilities based on development periods and project developers, and the spatial structure of the station changed from one development period to another. This led to changes in transfer routes and exit numbers. As a result, most of the first-time visitors were lost during transit or experienced an arduous transfer path. Against this backdrop, this study evaluated the emotional vocabulary and transit path distance of users through a wayfinding experiment in a specific section of Suwon station, where users are frequently confused and lost. This study provides an analysis framework for human environmental psychology and differences in transfer in two categories, familiarity and unfamiliarity, which are shown in the behavior of traveling to the destination in a complex space through wayfinding. Moreover, this will also become the basic data for spatial technology development for a better pedestrian environment. This study is meaningful as a suggestion for using empirical data being gathered by designating a site for the needs of city managers and citizens. In addition, it provides the specific spatial data necessary for spatial information design in the future, by extracting the patterns of movement and clearly distinguishing the visitors and users who are familiar with the space, and selecting them as participants in the experiment.

### 1.2. Literature Review

#### 1.2.1. Emotional Perception and Spatial Familiarity in Environment

The importance of the self-centered or information-storing factors during the processes of perceiving and recognizing space has been studied [20,21,22,23,24]. For example, in a study that focused on how visual stimuli were processed in space, participants who were not familiar with the environment learned it through guidance, whereas those who were familiar with it tended to rely on their long-term experiences [25]. In Demirbaş’s [26] study on the familiarity of spatial perception, it was revealed that personal characteristics and spatial familiarity are the important factors that influence spatial perception. In particular, spatial familiarity is mainly influenced by experience, spatial ability, meaning and expectation, and environmental complexity [27,28,29]. In this process, individual characteristics should be studied. For example, both the accuracy and time of perception in wayfinding and spatial orientation work improve depending on familiarity with the environment. Then, the complex environment layout becomes familiarized and easier to deal with [30]. In other words, as you become familiar with the environment, knowledge of objects or locations in the environment is increased compared to an unfamiliar environment [31].

Space familiarity can also vary depending on gender. In a study examining the impact in a real environment [30,32,33,34,35], men were more accurate and faster in becoming familiarized with space than women, and this difference was particularly evident in those who were not familiar with the environment. That is, it could be seen that the familiar group and males were faster and more accurate in becoming familiarized with space than the unfamiliar group and females. A study by Thorndyke and Hayes-Roth [10] classified the similarities or characteristics of familiar and unfamiliar groups. Both groups were accurate in a straight path, but the familiar group showed a better result in wayfinding. This can be interpreted that as one accumulates experience, spatial expression seems to become more user-centric. A study by Prestopnik and Roskos-Ewoldsen [36] showed that participants who are familiar with the environment are more accurate in their mental wayfinding tasks than those who are less familiar. While there was no gender difference in the navigation when participants were familiar with the environment, male dominance was clear when the participants were not familiar [35].

It has been verified through past research that the familiarity of humans affects the perception of spatial information, spatial sensibility, and behavior [18,37,38,39,40,41]. Human emotions are qualitative data with nonlinear characteristics, such as ambiguity and complexity. However, human sensibility also influences humans via the perception of space. This study presented emotional vocabulary for human emotions and qualitative data. In that way, the participants in the experiment could express their emotions as specific values in the process of wayfinding [42].

#### 1.2.2. Effecting Factors on Wayfinding Route

There have been studies on the factors influencing wayfinding, such as gender [35,43], age [20,34] and spatial familiarity [30,40].

In terms of age differences, older people have lower abilities in spatial exploration than younger people, such as evaluation of environmental knowledge and completion of learning tasks. Wayfinding is neurologically related to the hippocampus, whereas path learning is related to the caudate nucleus. Neurologically, age is correlated with a decreased volume of the frontal lobe and hippocampus. Mendez–Lopez, Fidalgo, Osma, and Juan [8] found that among the parameters that can influence wayfinding in a physical environment, gender difference significantly predicted effective wayfinding techniques. There was a difference between men and women in their self-reporting on space-oriented strategies [44] and the degree of confidence in their ability to solve spatial tasks.

Space familiarity influences the process of selecting and navigating a route in a space with complex movement patterns [45]. Millonig, Brändle, and Gartner [40] analyzed the behavioral patterns and routes taken by pedestrians through observations and surveys, and found that it is an interest in space that determines the route, not the selection of the shortest and most efficient route. Through a study on wayfinding, Guo and Loo [46] confirmed that it is the experience of using multiple routes that determines the route in space. In order to identify which factors influence the wayfinding behavior, studies have been conducted in various ways to observe the behaviors that occur when pedestrians search for a space and find their way in a complex space, [47]. Kang, Heo, and Hwang [48] conducted a survey and interview on foreign students on the factors of failures in wayfinding at subway transfer stations (Dongdaemun History and Culture Park Station, Express Bus Terminal Station, and Sadang Station). They found that the failures in wayfinding caused psychological reactions such as anger, embarrassment, resignation, and fear, leading to avoidance of visiting the space. Park, Oh, and Rhee [11] suggested data for inducing routes that can lead to reduction in traffic congestion and creating a comfortable subway station environment through a study on the route system. In addition, in order to increase the comfort and stability of walking, they also conducted a study looking at the amount of walking in the pedestrian routes of the subway transfer section to identify problems in the pedestrian routes. Lee, Kim, and You [49] examined changes in the width and separation of routes in order to see if the congestion caused by pedestrians in subway stations can be reduced. In addition, they also examined if users’ walking convenience and mobility could be improved. Jung, Chung, and You [50] conducted a follow-up survey and questionnaire survey to determine the effect of moving distance on path selection by checking the characteristics of selection of pedestrian routes in subway stations. They found that travel distance is the most important factor in selecting a route within a subway station, and it was revealed that station users use routes that can reduce travel distance by choosing the shortest travel distance. Through observing the perceptual psychological expression behavior of emotional cognition, the pattern type and time reduction for the wayfinding of the movement route can be seen as an evaluation of the human perception of a surrounding environment. This focus is considered a systematic observation in the behavioral science research methodology and lets the observed behavior of a small number of people be quantified [51]. The data extracted for the purpose of such a study can then predict the users’ psychology in the space by later simply observing their behavior. This study is thus significant in providing a research method that analyzes the mutual effects of the evaluation factors for psychological emotional factors along with the verification of studies of factors that influence pathfinding and behavioral observations.

## 2. Research Methods and Process

### 2.1. Experiments and Participants

#### 2.1.1. Experiment Contents and Process

Considering the psychological aspect of users who visited the train station, this study examined the perceived sense of user time based on transfer movement. In implementing the task of “A Study on Behavior Pattern Analysis for Reducing the Walking Time of Users” to establish a better environment and guide sign maintenance system at Suwon station, a questionnaire was given to users about the main exits and areas that are frequently confusing, and a behavioral observation experiment was conducted. The experiment investigated the walking process and perception of the participants while they were performing a Suwon station wayfinding task. The experimental space is actually a combination of two spaces, including a movement path from the second floor (referred to as Experimental space A) to the first basement floor (referred to as Experimental space B), which corresponds to the transfer space of the station.

In the experimental process, first, the participant was given an explanation about the entire experiment in the waiting space inside the station, and they moved to the starting point of the experiment. At this point, the following “vignette,” as a wayfinding mission, was given to the participant: “You just arrived at the station on KTX. You are about to board a city bus to get to the court in this city. After exiting the passage in front, go to Exit 13. However, please do not use your mobile phone or ask people around you”. The experiment started from the starting point of experiment space A and ended when they arrived at Exit 13, the destination of experiment space B. The time taken for the experiment was about 40 to 50 min, from the briefing to the point when the questionnaire given after the experiment was over.

#### 2.1.2. Participants

There were a total of 28 participants and the experiment period was a total of 5 days from 26 June 2020. Experimental participants with or without experience of visiting the test site were recruited separately. There was one researcher at the participant’s waiting area who gave the explanation of the experiment and post hoc surveys, and two researchers who videotaped and checked the movement paths. Due to the COVID-19 situation, the waiting time between experiments and the size and arrangement of the number of participants were adjusted, and all participants wore a face mask during the behavior experiments and surveys.

The participants were divided into familiar or unfamiliar groups regardless of whether they had visited the experiment site or not. The purpose of this study was to examine the differences in perception and movement patterns that could be seen in wayfinding at the test site depending on the degree of space perception. In the post-test questionnaire, residential area, number of visits to Suwon Station, and degree of familiarity with use of Suwon station were additionally investigated.

### 2.2. Data Analysis

#### 2.2.1. Affective Keywords of Emotional Evaluation

Spatial emotion was empirically evaluated. For empirical analysis, data collection was based on emotional vocabulary. After the wayfinding experiment was over, the participants were asked to evaluate the test site on an iPad using a total of 20 emotion affective keywords on a Google form.

In the evaluation of emotional perception, first we checked whether or not the experiment participant had previously paid a visit to Suwon station and was familiar with the experiment site. Second, the informational convenience and convenience factors of the wayfinding process and the information in the functional phase, as presented in the wayfinding experiment were confirmed. Third, an emotional vocabulary evaluation was conducted for the experimental location during the wayfinding process. The selection of emotional vocabulary was extracted from the emotional vocabulary questionnaire evaluation that was conducted in an existing pathfinding process experimental study [4,16,17,18,19]. The emotional affective keywords included “Clean, Leisurely, Pleasant, Organized, Bright, Wide, Open, Boring, Comfortable, Nervous, Tired, Tense, Exhausted, Uneasy, Crowded, Lively, Uncomfortable, Calm, Congested, and Uptight”.

For the selected emotional vocabulary, the semantic differential scale by Osgood [52,53] was used to measure the spatial sensitivity of the participants’ emotional vocabulary. In addition, the positive adjective 5-point scale was evaluated. Presenting the selected emotional vocabulary and its negative words at both extremes was an attempt to fully understand the degree of spatial sensibility generated in the process of finding one’s way [54]. SPSS was used to analyze the data in a T-test, factor analysis, and multidimensional scaling (MDS).

#### 2.2.2. Experimental Wayfinding Site

**Figure 1:**
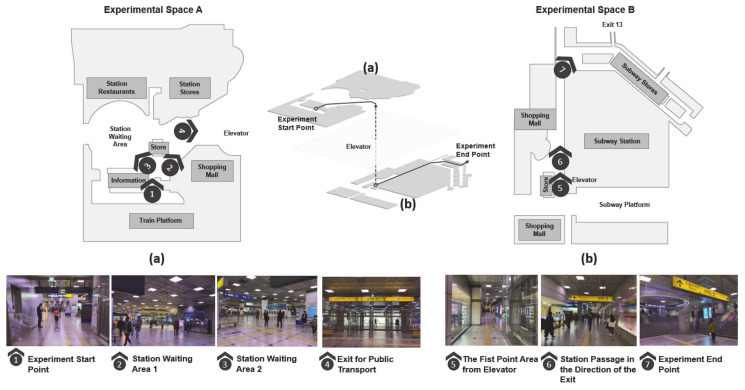
Transfer space for the experiment site: (**a**) Experimental space A: starting point on the second floor; (**b**) Experimental space B: this space located on the first basement level was the destination point.

Each participant was tracked with OSMO Pocket OT 110 during the experiment in order to trace the movement patterns during the wayfinding. For the sake of accuracy, the researcher separately traced the participants and marked their movement patterns on a drawing. After that, the recorded images and movement patterns were compared. Then, using AutoCAD, movement patterns from the starting point to the arrival point were recorded on a 800:1 scale drawing that represented experiment space A (second floor above ground) and experiment space B (first basement floor) for each experiment participant. The data on the movement patterns were used for analysis by calculating the actual distance using the dimensional scale. The transfer station, the test site, was recreated in the form of a diagram, a spatial map, and a spatial view (see Figure 1).

## 3. Results

### 3.1. Comparison of Emotional Affective Keywords in Evaluation

#### 3.1.1. Evaluation of Spatial Perception by Participant Group

In the post-experiment survey response analysis, participants (*n* = 28) were divided into those having experience (*n* = 16) or no experience (*n* = 12). Regarding familiarity with the test site space, they were familiar (*n* = 12) or not familiar (*n* = 16). For those who had visited the site, there was a slight difference in familiarity and unfamiliarity. There was a statistically significant difference in familiarity with the test site in the post hoc questionnaire compared to that of experiencing the space as a place of residence or work (χ^2^ = 17.173, *p* > 0.05).

After the experiment, a 5-point Likert scale questionnaire was used to record the emotional affective keywords for the test site. As a result of the questionnaire data analysis, there were more negative vocabularies for evaluating the corresponding space, with fewer positive keywords. The vocabulary for negative evaluation included “Nervous”, “Tired”, “Tense”, and “Uptight”, in that order. The vocabulary for positive evaluation included “Organized”, ”Comfortable”, and “Leisurely”, in that order. Participants were classified according to gender, age, and spatial familiarity, and the values showing significant differences in the responses of each group were organized in a table.

As shown in Table 1, the items of “Leisurely”, “Uneasy”, “Nervous”, “Exhausted”, and “Uptight” were statistically significant by gender (men (*n* = 14) and women (*n* = 14)) (*p* < 0.05), and “Tense” and “Tired” showed a significant difference (*p* < 0.10). For “Tense”, “Uneasy”, “Nervous”, “Exhausted”, “Tired”, and “Uptight” women had higher negative perceptions than men. As shown in Table 2, the 30–35 year old (*n* = 13) and 35–44 year old (*n* = 15) groups were statistically significant for the items “Tense”, “Uptight”, “Nervous”, and “Tired”. There was a significant difference for the “Exhausted” item (*p* < 0.10), and the “Congested” item was relatively high in the 40–44-year old group. As shown in Table 3, the “Wide” and “Crowded” items showed significant differences depending on whether the participants were familiar with the space (*p* < 0.05). The familiar group had a relatively high negative perception in the “Crowded” category. In the emotional vocabulary for negative perception, “Tired”, “Tired”, “Nervous”, “Exhausted”, and “Uptight”, perceptions were relatively similar, regardless of whether they were familiar with the space or not.

**Figure 2:**
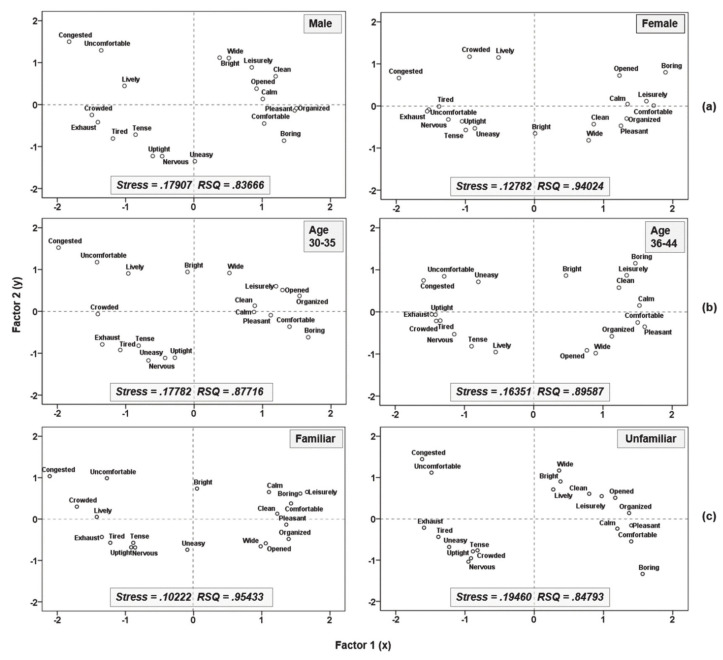
MDS (multidimensional scale) matrix applied to distances between emotional affectivity keywords of space: (**a**) emotional keyword scale according to gender (male–female); (**b**) emotional keyword scale according to age; (**c**) emotional keyword scale according to spatial familiarity (familiar–unfamiliar).

Similarly, space perceptions can be compared by putting perceived emotional keywords together. Therefore, space perception was visualized in two dimensions using a multidimensional scale (MDS) (see Figure 2). Participants were grouped for emotional keyword recognition according to gender, age, and space familiarity, and emotional keyword scale coordinates were prepared according to similarity. The more emotional space evaluation keywords were located on the right side of the coordinate factor 1 (x), and correspond to a more comfortable space; the more they are located on the left, the more uncomfortable they are. Depending on the distance level of the emotional keywords, factor 1 (horizontal axis) can be interpreted as reflecting the emotional factor of the comfort level. Factor 2 (vertical axis) shows positive vocabulary responses when it is positioned at the top, and negative keywords responses are shown at the bottom. Factor 2 (y) is interpreted as a space management factor.

For gender (see Figure 2a), it is the estimated distance between the coordinates of the emotional vocabulary in males (Stress = 0.17907, RSQ = 0.83666) and females (Stress = 0.12782, RSQ = 0.94024) (the relationship distance between keywords groups), which can be interpreted as an emotional evaluation. The groups of emotional affective keywords located on the *x*-axis and *y*-axis look similar, but depending on the gender, the vocabularies of “opened”, “wide”, “bright”, and “boring” are located on the right side of the *x*-axis, whereas along the *y*-axis they are on the opposite side for males (top) and females (bottom). The emotional evaluation of the management factor seems to differ according to gender. Depending on the age (see Figure 2b), there is a distinct difference in the distance of the coordinates of the emotional vocabulary between 30–35-year olds (Stress = 0.17782, RSQ = 0.87716) and 36–44-year olds (Stress = 0.16351, RSQ = 0.89587). In the 30–44 year old group, “opened”, “wide”, “organized”, and “pleasant” showed differences in the negative emotional evaluation of management factors. For spatial familiarity (see Figure 2c), there was a distinct difference in the distance of the emotional vocabulary coordinates between the familiar (Stress = 0.10222, RSQ = 0.95433) and unfamiliar (Stress = 0.19460, RSQ = 0.84793) groups. Having a distinct difference in the distance between emotional affective keyword coordinates can be interpreted as meaningful because the relationship between them is clear. In the spatial unfamiliarity group, the keyword “boring” is the most prominently located at the bottom of the management factor, and “uneasy”, “crowded”, and “nervous” are located at the bottom left of the emotional factor and management factor.

**Figure 3:**
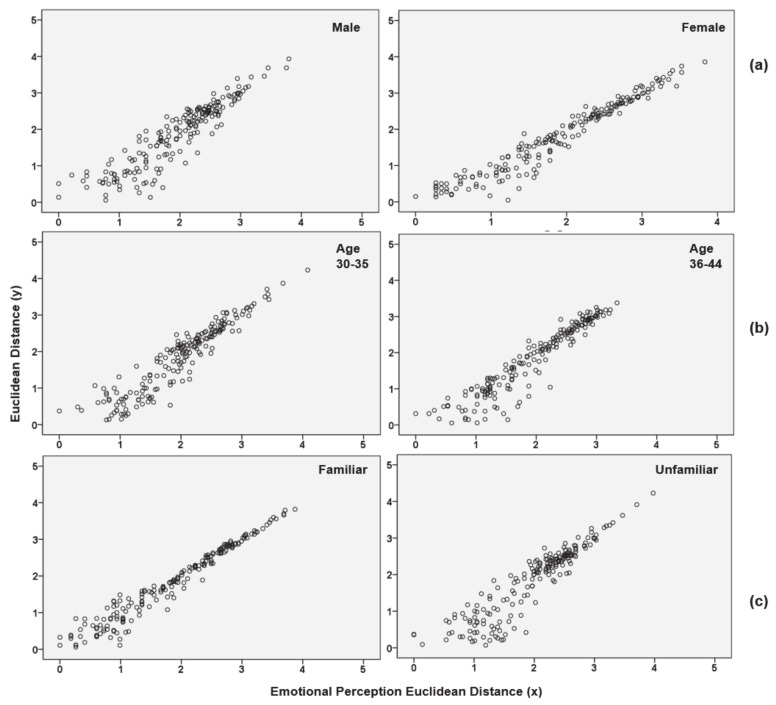
Scatter plot of linear fit from a Euclidian model: (**a**) gender difference; (**b**) group difference; (**c**) spatial familiarity difference.

Figure 3 is a scatter plot showing the fit between the actual Euclidean distance and estimated distance for the perceived values according to gender, age, and spatial familiarity. It seems that the multidimensional scale coordinate model of Figure 2 is a good fit because they are all diagonally distributed. In the gender group (Figure 3a), the diagonal distribution is more consistent in females than in males. While, in the age difference group (Figure 3b), the two subgroups show similar distributions. It appears that the scatterplot of the spatial familiarity group (Figure 3c) is more concentrated along the diagonal for familiar responses.

#### 3.1.2. Participant Group Positioning for Spatial Perception Factor Extraction

For the response to all emotional keywords items, principal component analysis and Varimax rotation were used for factor analysis. Factor analysis was conducted on the transfer experiences for which the factors were most clearly classified, resulting in three factors. The Kaiser Meyer Olkin (KMO) measure was 0.564, and the result of Bartlett’s sphericity test also showed a significance probability of less than 0.001, indicating that the factor analysis model was a good fit. In addition, the cumulative variance was 60.148%, showing a high explanatory power for the three factors. As components of each factor, the first factor had “Nervous”, “Tired”, “Uptight”, “Tense”, “Exhausted”, “Uneasy”, “Crowded”, “Leisurely”, and “Lively”, and was named “Emotional Factor” based on the vocabulary content classified in the first factor. The second factor had “Pleasant”, “Clean”, “Organized”, “Bright”, “Wide”, and “Open” and was named “Aesthetic Factor” based on the vocabulary contents classified in the second factor. The third factor had “Uncomfortable”, “Congested”, “Calm”, “Comfortable”, and “Boring”, and was named “Management Factor” based on the vocabulary content classified in the third factor (see Table 4).

**Figure 4:**
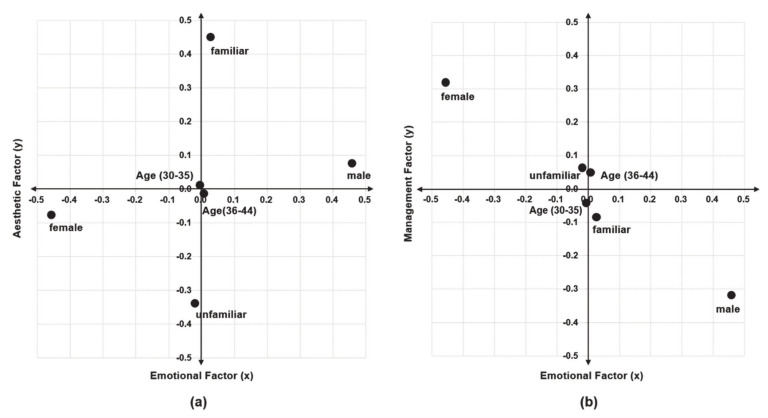
Positioning map on group differences according to factor axis: (**a**) coordinates of the Emotional Factor(x)–Aesthetic Factor(y) axis; (**b**) coordinates of the Emotional Factor(x)–Management Factor (y) axis.

Positions for each respondent group (gender, age, and spatial familiarity) were identified according to the coordinates of the three factors extracted in the spatial perception factor analysis (see Figure 4). The three factors (emotional factor, management factor, and aesthetic factor) were turned into groups of two factors, and the averages of those groups were compared on a positioning map. The characteristics of the factors that are statistically significant for each group in each positioning map are as follows. Figure 4a shows the evaluation of Emotional Factor (x)–Aesthetic Factor (y). In the emotional factor along *x*-axis (F = 7.202, *p* < 0.05), space evaluation by females was negative. In the aesthetic factor of spatial familiarity (F = 4.863, *p* < 0.05), the familiar and unfamiliar groups showed opposite results, with the unfamiliar group showing a high negative perception. Figure 4b shows the evaluation of Emotional (x)–Management (y) factors. Females had a negative evaluation on the emotional factor on the *x*-axis (F = 7.202, *p* < 0.05), and males and females were significantly different for the management factor (F = 3.058, *p* < 0.10). There was a difference in the groups of gender and spatial familiarity; in the emotional factor, the female and unfamiliar groups had negative evaluations. In addition, the male and familiar groups had negative evaluations in the management factor.

### 3.2. Movement Pattern Behavior in Wayfinding

#### 3.2.1. Differences in Distance in Wayfinding

As a result of calculating the walking distance of all participants in wayfinding, the average distance was 182.27 m (SD = 146.49 m) in Experimental space A, and 205.21 m (SD = 64.25 m) in Experimental space B. Compared to experimental space B, the deviations in the length of the movement patterns were greater in Experimental space A. In Experimental space A, the difference between the shortest movement pattern (59.04 m) and the longest movement pattern (662.56 m) was 603.52 m, which is about 10 times longer. The difference between the shortest movement pattern (154.48 m) and the longest movement pattern (441.28 m) in Experimental space B was 286.8 m, which is about three times longer. As for the entire paths in both spaces, the difference in distance between the participant with the shortest movement pattern (217.84 m) and the participant with the longest movement pattern (845.52 m) was 627.68 m, which is about four times longer.

Overall, familiar participants showed shorter movement patterns and travel times in both spaces (test spaces A and B). When the distances for movement pattern were compared based on familiarity, unfamiliar participants (241 m) in Experimental space A moved 137 m more than familiar participants (104 m). In Experimental space B, unfamiliar participants (229.3 m) moved 56.2 m more than familiar participants (173.1 m). On the second floor above the ground, unfamiliar participants moved a difference about twice as long as on the first basement floor.

#### 3.2.2. Frequency of Wayfinding Types in Experimental Sites

In wayfinding, pedestrians make various walking movements, and the representative movement patterns appearing on the second floor and the first basement floor were classified into types. In Experimental space A, the movement patterns were classified into “P1 = shortest distance”, “P2 = one turn”, “P3 = zigzag”, “P4 = one full circle”, and “P5 = return”, in that order. In Experimental space B, they were classified into “P1 = shortest distance”, “P2 = one turn”, “P3 = zigzag”, “P4 = one full circle”, and “P5 = turn around”. Unlike the “P1, P2, P3, and P4” types that had the same pattern in both spaces, “P5 = return” in Experimental space A was a case of returning to the starting point. In addition, “P5 = turn around” in Experimental space B refers to the type of movement pattern in which the participant got off the elevator, turned to the left, and went around the mall. The following diagram shows the movement pattern types and a table of frequencies according to the type of movement by floor.

In Experimental space A, the frequency of the shortest distance P1 movement pattern was significantly higher in the familiar group than in the unfamiliar group. In the unfamiliar group, none of the 16 participants followed the P1 movement pattern, but most of the 12 members in the familiar group walked along the P1 (*n* = 11) movement pattern. Conversely, the frequency in the unfamiliar group was in the order P2 (*n* = 7), P3 (*n* = 5), P4 (*n* = 3), and P5 (*n* = 1), except P1.

**Figure 5:**
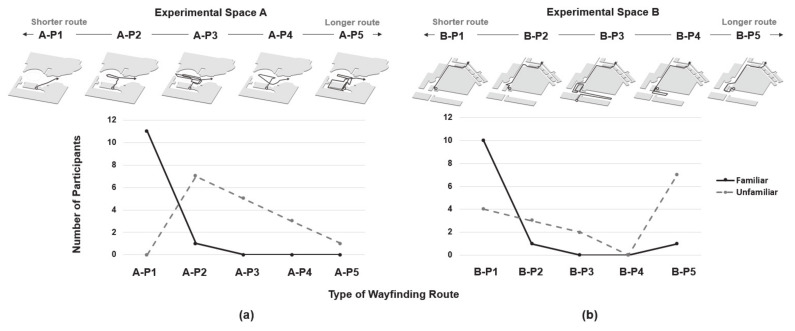
Extraction of movement patterns in wayfinding in an experimental environment (**a**) movement patterns in Experimental space A (A-P1–A-P5) and their frequencies; (**b**) movement patterns in Experimental space B (B-P1–B-P5) and their frequencies.

In Experimental space B, the frequency of the shortest distance P1 (*n* = 10) was overwhelmingly high in the familiar group; whereas the unfamiliar group had the highest frequency for P5 (*n* = 7). On the drawing, in the cases where the participants got off the elevator and failed to see the information sign, nearly half of them showed the pattern of going back to the other side of the destination at the crossroad due to a commercial building. Given that the ultimate goal of efficient wayfinding is to go from the starting point to the destination in the shortest distance, the frequency difference in the P1 movement pattern in Experimental space A and Experimental space B was not great in the familiar group; whereas, in the unfamiliar group, especially in Experimental space A, almost all participants had difficulty in wayfinding, and in Experimental space B, wayfinding was difficult for 75% of the participants (see Figure 5).

#### 3.2.3. Spatial Familiarity of Users and Elements of Interaction

For Experimental spaces A and B, the correlations of the main variables in the experiment participants, such as gender, age, whether they were near their residence or workplace, and whether they had experience with the space, were statistically analyzed. Two-way ANOVA was used to find the main effects of gender and familiarity on the length of movement patterns in Experimental space A and Experimental space B, and the interaction effects between the two variables. The results showed that the main effect of familiarity with the length of movement patterns was not significant. The main effects of experimental environment A (F = 6.212, *p* < 0.05), experimental environment B (F = 5.251, *p* < 0.05), and gender were also not significant.

Familiarity with the length of wayfinding was significant, and a post-hoc Bonferroni’s multiple comparison was performed for a more detailed analysis. In Experimental space A, the difference between familiar (155.4 m) and unfamiliar (228.03 m) was small in males; in females, the difference between familiar (67.27 m) and unfamiliar (257.61 m) was large; whereas, in Experimental space B, the difference was not very large between males (familiar, 173.84 m and unfamiliar, 244.77 m) and females (familiar, 172.54 m and unfamiliar, 209.41 m). In other words, the relationship between the length of movement patterns and familiarity depended on experimental space.

**Figure 6:**
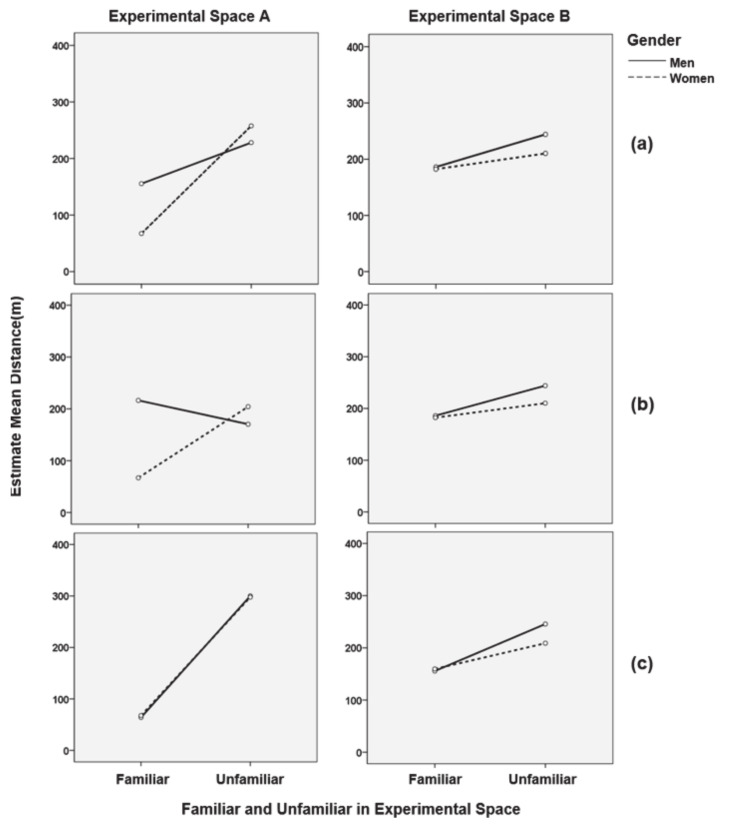
Statistical verification of the interaction effect between familiarity and the length of wayfinding movement length, gender, and the modulating effect of age: (**a**) interaction effect between familiarity with the length of wayfinding movement length and gender; (**b**) influence of age (30s) on the interaction between familiarity with the length of wayfinding movement length and gender; (**c**) influence of age (40s) on the interaction between familiarity with the length of wayfinding movement length and gender.

A three-way ANOVA was used to find out if age played any role in the interaction effect between familiarity and gender. The results showed that the interaction effect was not significant for familiarity, sex, and age in Experimental spaces A and B. Regarding the mean differences and interaction effects, in Experimental space A, there was a difference in the interaction between familiarity and gender in the 30s, but not in the 40s. In mean differences, the difference between familiar (216.334 m) and unfamiliar (170.36 m) in men in their 30s was not large, but in women, the difference was great between familiar (66.97 m) and unfamiliar (204.16 m). In their 40s, the values for familiar and unfamiliar groups were almost the same for both genders: males with familiar (64 m) and unfamiliar (300.12 m), and females with familiar (67.66 m) and unfamiliar (297.70 m). In their 30s, males in Experimental space B had familiar (186.03 m) and unfamiliar (244.10 m), whereas females had familiar (182.27 m) and unfamiliar (210.23 m). In their 40s, males had familiar (155.55 m) and unfamiliar (245.62 m), whereas females had familiar (159.56 m) and unfamiliar (208.80 m). The results of this analysis are summarized as follows (see Figure 6).

If their residence or workplace was in the test area, participants may be familiar with the surrounding traffic. Thus, the interaction between familiarity and locality was tested. Results showed that the main effects of familiarity and locality were not significant in Experimental space A. However, in Experimental space B, the main effects for familiarity (F = 12.561, *p* < 0.01) and locality (F = 4.349, *p* < 0.05) and their interaction effect (F = 4.682, *p* < 0.05) were all significant. In the mean comparison, the difference between familiar (67.32 m) and unfamiliar (283.9 m) of local residents in Experimental space A was large, and the difference between familiar (155.32 m) and unfamiliar (238.11 m) of non-local residents was not large. In Experimental space B, the difference between familiar (172 m) and unfamiliar (361.7 m) in local residents doubled, and the difference between familiar (174.6 m) and unfamiliar (220.48 m) in non-local residents was rather small.

**Figure 7:**
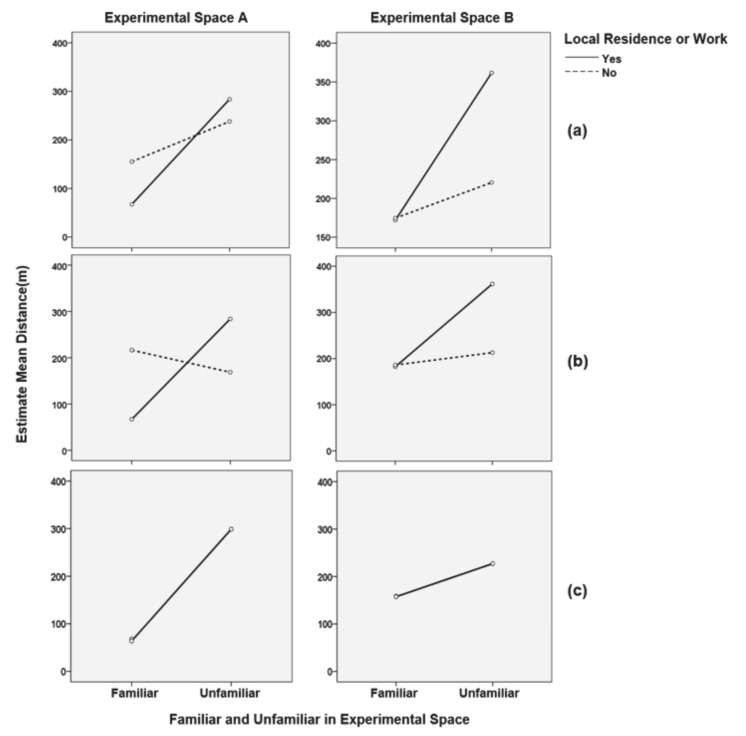
Statistical verification of interaction effects between familiarity on the wayfinding movement pattern length and locality (whether they were local residents or live close to workplace) and the role of age as a modulator. (**a**) interaction effect between familiarity and locality; (**b**) influence of 30s on the interaction between familiarity and locality; (**c**) influence of 40s on the interaction between familiarity and locality.

A three-way ANOVA was conducted to test whether age plays a role in the interaction between familiarity and locality. In experimental spaces A and B, there was no significant interaction among familiarity, locality, and age. Regarding mean difference and interaction effect, in experimental space A, there appeared to be a difference in the interaction between familiarity and gender in the 30s, but not in the 40s. For mean difference, the difference between familiar (66.97 m) and unfamiliar (283.9 m) was great for local residents in their 30s, but for non-local residents, the difference was not great with familiar (216.33 m) and unfamiliar (168.62 m). For those in their 40s, there was not much difference in familiarity, depending on whether they were local residents or not. In Experimental space A, local residents had a familiar (67.8 m), and non-local residents had a familiar (63.8 m) and unfamiliar (298.89 m), which was a big difference. In Experimental space B, local residents in their 30s had a longer length in unfamiliar (361.7 m) than in familiar (182.27 m), and non-local residents had a familiar (186.03 m) and unfamiliar (212.78 m). Therefore, the difference was larger among local residents. Local residents in their 40s had a familiar (158.30 m), while non-local residents had a familiar (157.45 m) and unfamiliar (227.21 m). In conclusion, in Experimental spaces A and B, there was a big difference in walking distance depending on whether or not they were familiar with the area, even for local residents in their 30s; whereas, for those in their 40s, the slope was the same in the familiar and unfamiliar groups, regardless of whether they were local residents (see Figure 7).

As a result of this analysis, there was a limit of 28 experimental participants who participated in the actual analysis when deriving the results. This limited number of people were classified into three groups (familiarity, gender, and age), and the interaction effects were visualized as a small sample size to analyze the effects clearly. In the statistical analysis, only significant values for the main effect were mentioned. In addition, it was meaningful to provide clues about the entire frame with regard to the experimental method and also provide basic data for the design of the space and improvement of the queuing arrangement, by providing factors that influence user characteristics as observed during the route wayfinding process.

## 4. Conclusion and Discussion

Wayfinding is a very natural behavior in our daily life. In wayfinding, our perceptions and actions interact, allowing us to start from the starting point and reach our destination. Understanding how we perceive and behave in a pedestrian environment is of great significance in designing a space that is easy to navigate. In particular, for important means of transportation, such as trains and subway stations, it is even more important to create a comfortable space in places where thousands or tens of thousands of people travel every day. To say that you are familiar with the environment suggests that the space is well-planned in terms of spatial information and structural provision. Studies have been conducted on various research methods for wayfinding. In this study, types of movement pattern and emotional evaluation were analyzed to extract influence factors according to the types of user.

This study examined how familiarity with space affects emotional evaluation of the space. The group familiar with the space complained of more discomfort, recognizing the problems, than the unfamiliar group in the emotional evaluation questionnaire. For example, in terms of emotional factors, emotional evaluation turned out to be high, such as “too much traffic”, “not affordable”, and “too complicated for use”. It seems that although the users were familiar with the space, they had a negative evaluation, shown as degrees of discomfort when using the transfer. Given that, it seems that both familiar and unfamiliar participants in the emotional perception evaluation tended to be biased toward negative emotional factors in both emotional, aesthetic, and usable emotional evaluations, and in particular, the familiar group showed a more negative emotional psychology. As in a study showing the tendency to perceive direction and accuracy for directions according to how familiar the environment is [30,31,55] Chalmers and Knight, 1985), when the environment is learned through direct experience, the knowledge of space will be handled from more flexible and various perspectives [25].

The difference between familiar and unfamiliar groups was clear in the observation of movement patterns. As a result of examining the types of movement pattern in Experimental spaces A and B through frequency analysis, most members of the familiar group recognized the shortest movement pattern in reaching the destination, whereas the unfamiliar group had a high frequency of a wandering movement patterns, and the difference in travel distance between the two groups was more than double. In addition, regarding the deviations in average distance in movement patterns, there were more deviations in movement pattern in the unfamiliar group in Experimental space A than in Experimental space B. As a result of analyzing the type of movement patterns in the unfamiliar group while wayfinding, it seems that the guidance system available in the space is not helpful for transfer. As noted in Prestopnik and Roskos–Ewoldsen [36] and Iachini, Ruotolo, and Ruggiero [35], knowledge is handled from more flexible and various perspectives when the environment is learned primarily through direct experience in wayfinding, depending on gender and familiarity. Likewise, familiarity with the space led to rapid movement patterns in emotional evaluation and space wayfinding. From the results of analyzing the type of traffic of the inexperienced group in the process of finding their way, it was confirmed that the guide system at Suwon station was not helpful for transfer.

In conclusion, this study is meaningful in that it made an empirical analysis of behavioral observations and an investigation of how familiarity with space affects cognitive behavior through an experiment called wayfinding. A train station is a space that serves as both departure and arrival points for the city, and as a transfer space connecting departure and arrival. Therefore, depending on how the local facility environment and guide sign maintenance system are planned and provided to the users, they can more or less easily recognize the directions for wayfinding. This implies that users can eventually form a positive emotional system of familiarity with the environment. This study conducted an experiment to study the differences and characteristics of people who are psychologically familiar or unfamiliar with human environmental psychology and movement patterns in the process of starting from a certain point and arriving at a destination on their first visit. Therefore, this study is meaningful in that it provided quantitative data for a better environment and system construction plan for confusing transfer areas, and it is possible to use this study as basic data for improving the pedestrian environment.

On the other hand, there was a limitation when deriving the results due to the number of participants in the wayfinding experiment. However, it makes sense to frame the experimental method and analysis in the result. By using the participatory group diversification process in future, spatial technology development data for the walking environment can be better accumulated and used significantly as valuable data for improving the walking environment overall.

## Figures and Tables

**Table 1:** Emotional affective keywords evaluation according to gender.

Emotional Affective Keywords	Gender	Mean	Standard Deviation	*t*	*p*
Leisurely	Male	2.57	0.76	−2.749 *	0.011
	Female	3.29	0.61		
Tense	Male	2.64	1.01	1.800 ^†^	0.083
	Female	2	0.88		
Uneasy	Male	2.79	1.05	2.506 *	0.019
	Female	1.93	0.73		
Nervous	Male	2.79	1.05	2.666 *	0.013
	Female	1.86	0.77		
Tired	Male	2.36	1.01	1.743 ^†^	0.093
	Female	1.79	0.7		
Exhausted	Male	2.57	1.02	2.449 *	0.021
	Female	1.71	0.83		
Uptight	Male	3.21	0.8	3.944 *	0.001
	Female	2.07	0.73		

* *p* < 0.05, ^†^ *p* < 0.10.

**Table 2:** Emotional affective keywords evaluation according to age.

Emotional Affective Keywords	Age	Mean	Standard Deviation	*F*	*p*
Tense	30–34	2.23	0.927	5.228 *	0.013
	35–39	2.9	0.738		
	40–44	1.4	0.894		
Uptight	30–34	2.46	0.967	6.238 *	0.006
	35–39	2.8	0.789		
	40–44	1.2	0.447		
Nervous	30–34	2.23	1.013	4.716 *	0.018
	35–39	2.9	0.738		
	40–44	1.4	0.894		
Tired	30–34	2	0.913	5.475 *	0.011
	35–39	2.6	0.699		
	40–44	1.2	0.447		
Exhausted	30–34	2.15	1.144	2.157 ^†^	0.137
	35–39	2.5	0.85		
	40–44	1.4	0.548		

* *p* < 0.05, ^†^ *p* < 0.10.

**Table 3:** Emotional affective keywords evaluation according to spatial familiarity.

Emotional Affective Keywords	Spatial Familiarity	Mean	Standard Deviation	*t*	*p*
Wide	Familiar	3.17	0.72	2.609 *	0.015
	Unfamiliar	2.31	0.95		
Crowded	Familiar	1.75	0.62	−2.086 *	0.047
	Unfamiliar	2.38	0.89		

* *p* < 0.05.

**Table 4:** Factor Analysis for Emotional Affective Keywords.

Emotional Affective Keywords	Factor 1	Factor 2	Factor 3
Nervous	0.916	0.028	0.009
Tired	0.884	−0.061	−0.035
Uptight	0.865	0.042	−0.097
Tense	0.833	0.072	−0.072
Exhausted	0.767	−0.102	−0.277
Uneasy	0.622	0.083	−0.558
Crowded	0.606	−0.403	0.171
Leisurely	−0.551	0.509	0.244
Lively	0.418	0.04	0.150
Pleasant	−0.022	0.82	0.316
Clean	−0.148	0.816	0.082
Organized	−0.042	0.778	−0.336
Bright	−0.068	0.628	0.130
Wide	0.089	0.606	0.113
Open	0.13	0.535	0.013
Uncomfortable	−0.051	0.042	−0.791
Congested	−0.103	−0.184	−0.753
Calm	−0.226	0.54	0.649
Comfortable	−0.121	0.391	0.635
Boring	−0.071	−0.017	0.553
Eigenvalue	5.02	3.922	3.087
commonality proportion of variance (%)	25.101	19.612	15.435
cumulative proportion of variance (%)	25.101	44.713	60.148

Kaiser Meyer Olkin (KMO) = 0.564, Bartlett’s χ^2^ = 377.501 (*p* < 0.001).

## Data Availability

Data sharing not applicable to this article.
